# 2-Dimensional Ti_3_C_2_T_x_/NaF nano-composites as electrode materials for hybrid battery-supercapacitor applications

**DOI:** 10.1038/s41598-024-52280-4

**Published:** 2024-01-18

**Authors:** M. Bilal Riaz, Danish Hussain, Saif Ullah Awan, Syed Rizwan, Sana Zainab, Saqlain A. Shah

**Affiliations:** 1 Department of Electrical Engineering, College of Electrical and Mechanical Engineering, National University of Sciences and Technology (NUST), Islamabad, 44000 Pakistan; 2 Department of Mechatronics Engineering, NUST College of Electrical and Mechanical Engineering, National University of Sciences and Technology (NUST), Islamabad, 44000 Pakistan; 3 Physics Characterization and Simulation Lab (PCSL), Department of Physics, School of Natural Sciences (SNS), National University of Sciences and Technology (NUST), Islamabad, 44000 Pakistan; 4 Department of Physics, Forman Christian College (University), Lahore, Pakistan

**Keywords:** Electrochemistry, Energy, Materials chemistry, Chemical synthesis, Energy storage, Condensed-matter physics, Materials for devices, Materials for energy and catalysis, Nanoscale materials, Structural materials, Nanoscale devices, Nanoscale materials, Applied physics, Chemical physics, Condensed-matter physics, Quantum physics, Chemical engineering, Electrical and electronic engineering, Energy infrastructure

## Abstract

The increasing global demand for energy storage solutions has spurred interest in advanced materials for electrochemical energy storage devices. Transition-metal carbides and nitrides, known as MXenes, are characterized by remarkable conductivity and tunable properties, They have gained significant attention for their potential in energy storage applications. The properties of two-dimensional (2-D) MXenes can be tuned by doping or composite formation. We report a novel Ti_3_C_2_T_x_/NaF composite prepared via a straightforward hydrothermal process for supercapacitor electrode applications. Three composites with varying NaF concentrations (1%, 3%, and 5%) were synthesized under similar conditions. Structural characterization using X-ray diffraction (XRD) and scanning electron microscopy confirmed the successful formation of the composites, whereas distinct shifts in XRD peaks and new peaks revealed the presence of NaF. Electrochemical performance was evaluated by cyclic voltammetry, galvanostatic charging-discharging, and electrochemical impedance spectroscopy. The composites exhibited pseudo-capacitive behavior with reversible redox reactions during charge and discharge cycles. Specific capacitance of 191 F/g at scan rates of 2 mV/s was measured in 1 M KOH. Electrochemical impedance spectroscopy revealed an escalating impedance factor as NaF content increases within Ti_3_C_2_T_x_. This study underscores the versatile energy storage potential of Ti_3_C_2_T_x_/NaF composites, offering insights into their tailored properties and behavior.

## Introduction

The rising energy demands and depletion of fossil fuel resources have attracted researchers to seek more creative and durable energy solutions^1^. Energy storage devices (ESD) are for various energy storage technologies such as wind and solar. ESDs store energy in various forms, including electrochemical, kinetic, pressure, potential, electromagnetic, chemical, and thermal. Electrochemical energy storage is one of the leading energy storage mediums, and it includes various devices such as batteries, fuel cells, standard capacitors, supercapacitors etc^2^. Factors such as energy density, power density, storage capacity, charge–discharge time/charging cycles, heat sensitivity, and maintenance/operating cost are important criterion used to select an ESD. Recently, supercapacitors have gained significant attention due to their higher power densities than batteries and higher energy densities than ordinary capacitors. SC can be categorized in two types: (1) electrical double-layer capacitors (EDLC), store charge electrostatically and no charge transfer occurs between the electrode and the electrolyte. Ions diffuse through the electrolyte to the electrodes because of the potential difference. (2) pseudocapacitors, the charge transport between the electrode and the electrolyte allows for electrochemical energy storage because of the redox reactions between the electrode and the electrolyte. In contrast to ELDCs, they have a larger specific capacitance and energy density because of reversible Faradic reactions at the electrode surface followed by charge transfer^3^, however their self-discharging process is their main limitation^4^.

Transition-metal carbides and nitrides (MXenes), are used as electrochemical energy storage materials because of their outstanding electrical conductivity, hydrophilic surface, excellent flexibility and tunable properties^5,6^. MXene are etched from their precursor M_n_ _+ 1_AX_n_ phases (where M is an early transition metal; A represent group A element and X is C and N) by selective etching of A phase which is mostly Al or Si. MAX powder mostly exhibit metallic or ceramic properties^7^. After selective etching of MAX powder, high quality MXene is obtained from supernatant post centrifugation and washing^8^. In the Ti_3_C_2_T_x_, T_x_ denotes surface terminated species, such as O, OH, and F groups^9^. MXenes have this advantage over other two dimensional materials that it can easily tune its properties and form composites with other materials owing to these surface terminations^10,11^. Mostly MXenes exhibit metallic behaviour but some MXenes gives semiconducting behaviour too^12^. Previously, various elements have been tested with Ti_3_C_2_T_x_, either in the form of doping or composite. Wen et. al. group had reported nitrogen^13^ and Sulphur^14^ doped Ti_3_C_2_T_x_ and clay inspired MXenes, previously^15^. Introduction of a foreign atom into MXene layers, can replace M or X atom to form stable bonds^16,17^. It is worthy to note that MXenes can easily form composites with other materials such as polymers, oxides, and carbon nanotubes, which further provides an effective way to tune the properties of the material for various applications such as energy storage^18,19^. For example, Sulfur decorated Ti_3_C_2_T_x_ showed specific capacity of 135 mA/g at 2 A/g current density making it well suitable for sodium ion batteries^20^. CuS decorated MXene exhibited a specific capacity of 169.5 C/g at current density of 1 A/g^21^. Under the current density of 0.1 A/g, specific capacity of 65 mAh/g was achieved for MnO_2_-MXene composite^22^. More importantly, three dimensional composite of MXene with porous carbon has proved to be an excellent host for sulfur in lithium sulfur batteries^23^. On the other hand, Sn^4+^ decorated MXene exhibited promising properties as an anode with a high reversible specific capacitance of 635 mAh/g at 0.1 A/g current density^24^. Zheng et al. prepared Au nanoparticles decorated MXene nanosheets with a specific capacitance of 278 F/g at 5 mV/s and 95% of cyclic stability after 10,000 cycles^25^. It was noted that polypyrrole particles acts as spacer preventing the restacking of MXene nanosheets, while contributing to higher capacitance of the hybrid material^26^. The phosphorus doped Ti_3_C_2_T_x_ with P–O and P–C bonds support rapid ion transfer into electrode thus provide a capacitance of 476.9 F/g^27^. Flexible nitrogen-doped carbon nanotube (N-CNT)/Ti_3_C_2_T_x_ (MXene)/polyacrylonitrile (PAN) nanocomposite films showed a high specific capacitance of 446.18 F/g at 5 mV/s ^28^. Three-dimensional (3D) hybrid porous aerogel composed of sulfur and nitrogen doped reduced graphene oxide and MXene (S,N-rGO@MXene) resulted in a specific capacitances of 85.4 F/g and 88.9 F/g, respectively^29^. Carbon-coated Fe_3_O_4_ nanoparticles were deposited on MXene nanosheets and the nanocomposite exhibited a specific capacity of 231.5 mAh/g even after 200 cycles^30^. MnO_2_-MXene-CNT fibers demonstrated a capacitance of 371.1 F/cm^3^ in three-electrode system^31^.

A hybrid supercapacitor-battery^39–42^ combines the features of both supercapacitors and batteries in a single device. Traditional batteries and supercapacitors have their respective limitations, such as low power density in batteries and low energy density in supercapacitors. The integration of these two technologies in a hybrid device overcomes these limitations, resulting in a system with significantly increased energy storage capacity (Es), enhanced power density (Ps), quick charging/discharging capabilities, and an extended cyclic lifespan. Hybrid battery-supercapacitor devices, also known as hybrid energy storage systems (HESS), offer a compelling combination of high power and energy density. By integrating batteries and supercapacitors, these hybrid systems strike a balance between the energy storage capacity of batteries and the rapid charge–discharge capabilities of supercapacitors. This balance is particularly advantageous for applications requiring bursts of high power and overall energy storage. Notably, the long cycle life of supercapacitors contributes to the extended durability of the hybrid system. Furthermore, the fast charging and discharging capabilities, temperature robustness, and enhanced efficiency make these devices well-suited for various applications, including electric vehicles, renewable energy systems, and portable electronics. The synergy between batteries and supercapacitors in hybrid devices not only maximizes performance but also extends the overall life of the energy storage system, showcasing their significance across diverse technological applications.

In this work, we investigate the influence of Ti_3_C_2_T_x_/NaF nano-composite to make an eledore for hybrid batter-supercapacitor device using the essential electrolyte KOH. First, Si was etched from Ti_3_SiC_2_ MAX powder^32^, which was further delaminated to create spacing between the layers of Ti_3_C_2_T_x_ nanosheets. Ti_3_C_2_Tx/NaF composite with concentrations ranging from 1 to 5% were prepared using the hydrothermal method. The material was used to fabricate the electrode using Ni foam as substrate, and XRD, SEM and EDX were used for analysis of the nanocomposite material. Electrochemical testing was used to observe specific capacitance, coloumbic efficiency and cyclic stability. GCD curves are to determine the specific capacitance, energy, and power density. Electrochemical Impedance Spectroscopy (EIS) was utilized to measure the electrical conductivity of the electrode material.

## Materials and methods

### Materials

Hydrofluoric acid (HF 48%), Hydrogen peroxide (H_2_O_2_ 35%), Tetramethylammonium hydroxide (TMAOH), Nickel foam was purchased from Sigma Aldrich and Co.

### Synthesis of Ti3C2 MXene 

**Figure 1:**
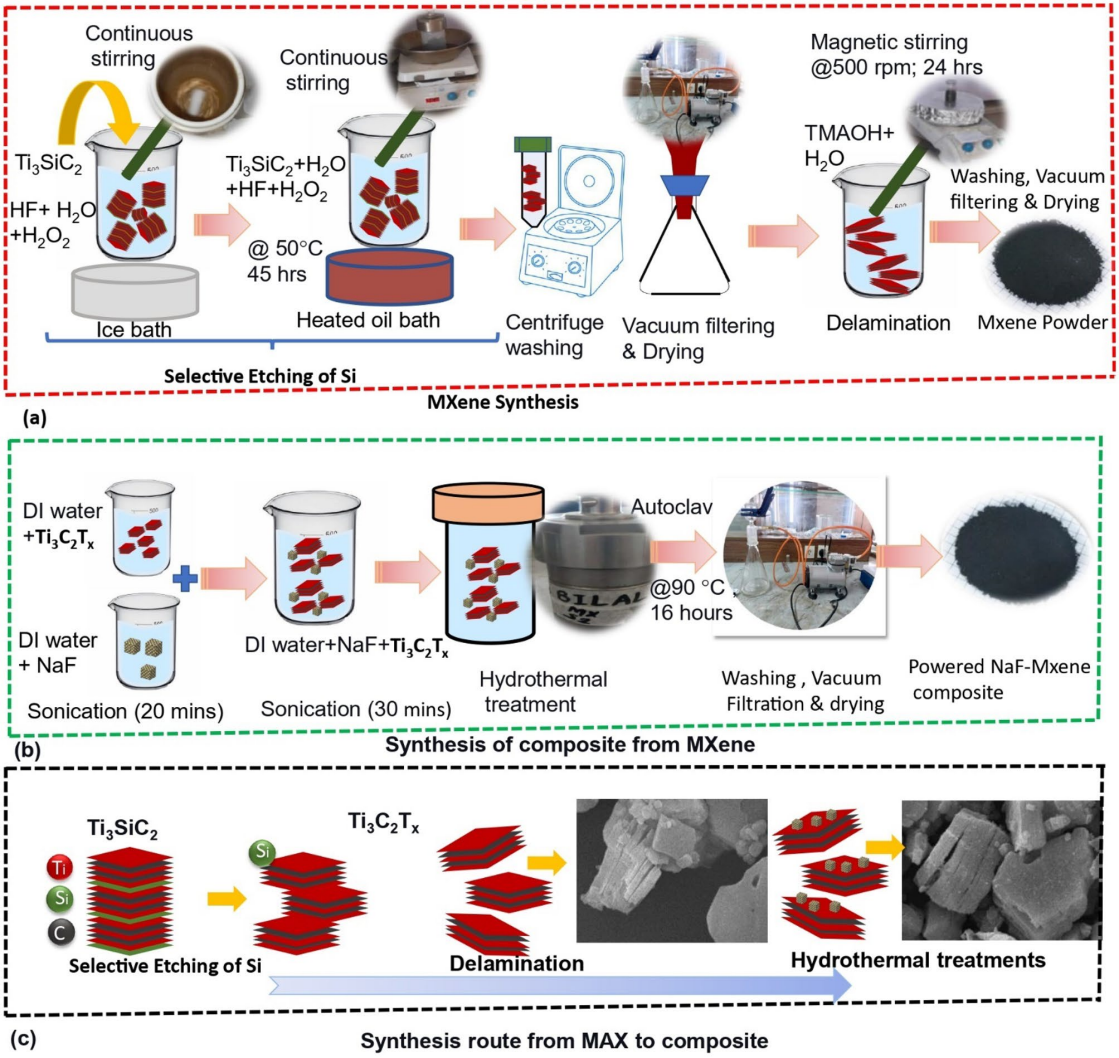
(**a**) MXene synthesis. (**b**) Composite synthesis. (**c**) Overall synthesis to obtain composite.

As shown in Fig. 1a–c, MXene (Ti_3_C_2_T_x_) was obtained by selective etching of Si from Ti_3_SiC_2_ using a straightforward oxidant-assisted method similar to one reported earlier^33^. The process begins by mixing 45 mL of diluted hydrofluoric acid (HF) with 5 mL of hydrogen peroxide (H_2_O_2_). (Caution: Hydrofluoric acid is an extremely corrosive substance, and it is of paramount importance to be fully cognizant of the associated risks and adhere to stringent safety protocols when working with HF and chemicals that generate HF).

Hydrogen peroxide is added to accelerate the etching as titanium and carbide in the MAX phase have a strong bond so Si is difficult to etch with HF alone. The prepared solution was carefully placed in an ice bath under continuous stirring for 20 min and 3 g of MAX powder was gradually introduced into the solution over time span of 20 min. The solution was kept on continuous stirring for 2 h at low temperature. Following this, the container was moved to a preheated silicone oil bath (at 50 °C) and was constantly stirred for 45 h at 500 rpm using a Teflon magnetic stirrer. This process facilitates separation Si from Ti_3_SiC_2_ and 2D structure is obtained. Subsequently, the obtained Ti_3_C_2_ was washed in DI water via centrifugation at 5000 rpm for multiple cycles to neutralize the pH and remove H_2_O_2_ and HF. Then the solution was vacuum-filtered using 0.2 μm filter paper, and the resulting powder was dried overnight in a vacuum oven set at 60 °C. The MXene powder was subsequently subjected to delamination using tetramethylammonium hydroxide (TMAOH). The process involves soaking of MXene powder in TMAOH, followed by dilution with water, and magnetic stirring at 500 rpm for 24 h. Afterwards, the pH was neutralized by washing using a centrifuge machine at 5000 rpm while continuously monitoring the pH. Additionally, during this process any remaining contaminants are cleaned as well. Then the solution was subjected to vacuum filtration using 0.2 μm filter paper and subsequently dried overnight in a vacuum oven at 40 °C.

### Synthesis of NaF-MXene composite

The synthesis of the NaF-MXene composite via a facile hydrothermal method was executed with meticulous attention. The procedure involves MXene dispersion in deionized water at a ratio of 20 mg/30 mL through 20 min of sonication, followed by a similar treatment of NaF dispersion (0.2 mg/20 mL) to ensure uniform distribution. These individual dispersions were subsequently amalgamated in a Teflon beaker and further sonicated for 30 min to achieve homogeneity. The resultant solution was then hydrothermally treated within a Teflon-lined autoclave at 90 °C for 16 h. Then the composite solution was vacuum filtrated and subsequently dried in a vacuum oven to eliminate residual solvents and water. The synthesis protocol was replicated to yield three distinct types of composites with NaF concentrations of 1%, 3%, and 5%, hereafter, referred as composite 1, 2 and 3, respectively.

### Electrode fabrication

**Figure 2:**
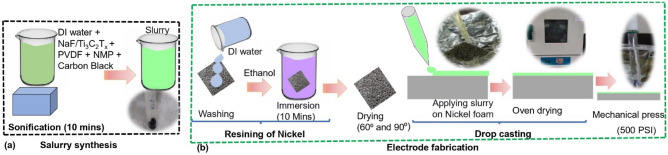
Electrode fabrication process, (**a**) Slurry synthesis process, (**b**) Electrode fabrication process.

The electrode fabrication process involves a sequence of precise steps as shown in Fig. 2a–b. Initially, the nickel foam (1 cm^2^) was meticulously cleaned, starting with a rinsing in DI water, followed by a 10 min through immersion in ethanol. Then the nickel foam was dried, at 60–90 °C until it was fully dried. For electrode fabrication slurry was formulated using precise proportions: 80% active material (Ti_3_C_2_T_x_/NaF), 10% polyvinylidene difluoride (PVDF) binder, and 10% carbon black, all suspended in N-methyl pyrrolidone (NMP) solvent. After 10 min of sonication, the slurry was drop-casted on the cleaned Ni Foam substrate. Then it underwent an overnight drying process in a vacuum oven and was subsequently mechanically compressed using a hydraulic press machine under 500 PSI pressure for five seconds.

### Characterization

Structural analysis of the Ti_3_C_2_T_x_ and Ti_3_C_2_Tx/NaF was performed using an X-ray diffractometer (DRON 8, BRUKER UNITED STATES) equipped with Cu Kα radiation as the X-ray source. Scanning Electron Microscopy (SEM), 6490A, JEOL Japan and energy dispersive X-ray spectroscopy (EDX) were used to study morphology and elemental distribution of the prepared samples. Gamry potentiostat was used to determine electrochemical properties. The choice between a two-electrode and a three-electrode system depends on the specific requirements of the electrochemical experiment. Two-electrode systems are simpler and suitable for basic measurements, while three-electrode systems offer more precise potential control and are preferred for advanced studies and applications requiring high accuracy. Initially three electrodes set up was used for cyclic voltammetry (CV), galvanostatic charge–discharge (GCD), and electrochemical impedance spectroscopy (EIS) measurements with composite material coated Ni foam as working electrode, Platinum wire as counter electrode and Ag/AgCl as reference electrode. The fabricated electrode was used as the working electrode. The chosen electrolyte was a 1 M KOH solution and the specific capacitance was measured as:-

1 $$C = \frac{1}{{2 \times s \times Vo \times \frac{dv}{{dt}}}}\mathop \int \limits_{{v_{i} }}^{{v_{f} }} I\left( V \right)dv$$

where I is the load current, V_o_ is the initial potential in the cyclic voltammograms, m is the mass of active material in the working electrode, v is the scan rate, and V is the potential window. The discharge time is obtained from the GCD curve, whereas energy and power density can be calculated from the discharge time using the following Eqs. (2) and (3).

2 $${\text{E}} = \frac{1}{2} \times {\text{ C}} \times \left( {{\Delta V}} \right)^{2} \times \frac{1}{3600}$$

3 $${\text{P}} = \frac{{\text{E}}}{{\text{t}}} \times 3600$$

In comparison of three electrodes, a dual-electrode setup is employed for conducting CV, GCD and EIS measurements. This configuration allows for a comprehensive analysis of electrochemical processes. In this system, two electrodes are strategically positioned within the electrochemical cell. The two-electrode studies in evaluating the electrode materials' real-time performance in practical device configurations. The working electrode serves as the site where the electrochemical reactions of interest occur, while the counter electrode facilitates the completion of these reactions by providing a pathway for electron transfer. This tandem electrode arrangement ensures the reliability and accuracy of the measurements. Cyclic voltammetry involves applying a potential sweep to the working electrode while monitoring the resulting current. Galvanostatic charge–discharge measurements maintain a constant current between the electrodes, providing insights into the energy storage or conversion capabilities of the system. Electrochemical impedance spectroscopy, on the other hand, examines the impedance response over a range of frequencies, offering valuable information about the system's kinetic and transport processes. The utilization of a two-electrode system in these techniques allows for a more comprehensive understanding of electrochemical behavior and performance in various applications, such as energy storage devices.

## Results and discussion

### X-ray diffraction analysis (XRD)

**Figure. 3:**
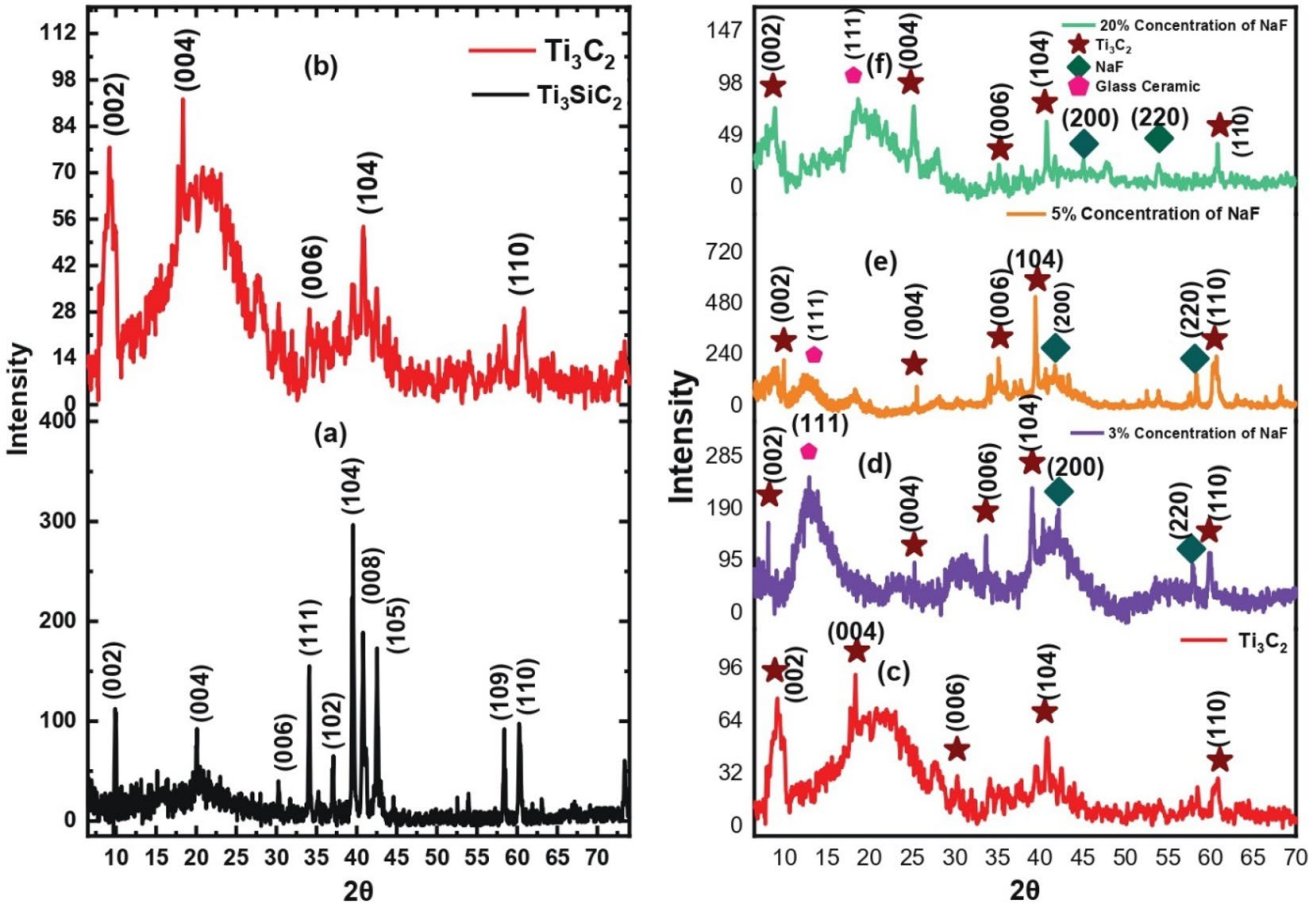
(**a**) The X-ray diffraction pattern of Ti_3_SiC_2_ Max phase. (**b**) The X-ray diffraction pattern of Ti_3_C_2_. The X-ray diffraction patterns of; (**c**) Ti_3_C_2_, (**d**) Composite 1, (**e**) Composite 2, (**f**) Composite 3.

The XRD patterns of commercial Ti_3_SiC_2_ MAX powder and the derived MXene were measured. Figure 3a shows XRD pattern of commercial Ti_3_SiC_2_ MAX powder, which has noticeable peaks at specific angles, such as 2θ = 9.10°, 20.05°, 31.30°, 34.50°, 37.70°, 39.50°, 41.30°, 42.50°, 58.40°, and 60.250°. These peaks correspond to distinct crystallographic planes inherent in the Ti_3_SiC_2_ structure. Figure 3b shows the shifted peaks for MXene at 2θ = 9.950°, 18.350°, 30.20°, 40.80°, and 60.85°. In the MXene several peaks were eliminated, whereas some peaks exhibited shifts compared to the initial pattern. The shift indicates increased interlayer spacing, while broadening of the (002) peak is due to Si layer substitution with functional groups (T_x_), signifying exfoliation and delamination^34^. Moreover, the presence of a minor peak along the (104) plane in the MXene pattern suggest minute amount of silicon (Si) within the resulting Ti_3_C_2_Tx MXene material.

Figure 3c presents the XRD patterns of pristine Ti_3_C_2_T_x_ sample. The XRD pattern of Ti_3_C_2_T_x_/NaF composite (composite 1) displays sharp peaks at 2θ = 8.12°, 25.28°, 33.68°, 39.09°, and 59.82° (see Fig. 3d) with few shifted to the left. The existence of additional peak at 2θ = 57.88° with the plane (220) suggests the impregnation of NaF into the layers and onto the surface of Ti_3_C_2_. Figure 3e displays sharp peaks centered at 2θ = 9.96°, 25.54°, 35.12°, 39.46°, 60.68° in the XRD pattern of the composite 2. The intensity of the peaks increases with the increasing doping concentration; a new NaF peak can be seen at 2θ = 58.32°. The XRD pattern composite 3 is shown in Fig. 3f. The patterns have distinct peaks at 2θ = 8.95°, 25.20°, 35.150°, 40.80°, and 60.85°, which are slightly shifted from left to right. The shift might reflect of incorporation of NaF within the composite structure. Overall, XRD results with left shifted peaks and peaks for NaF at certain angles confirm the formation of the composite.

### Morphological and structural analysis of NaF-MXene composite

**Figure 4:**
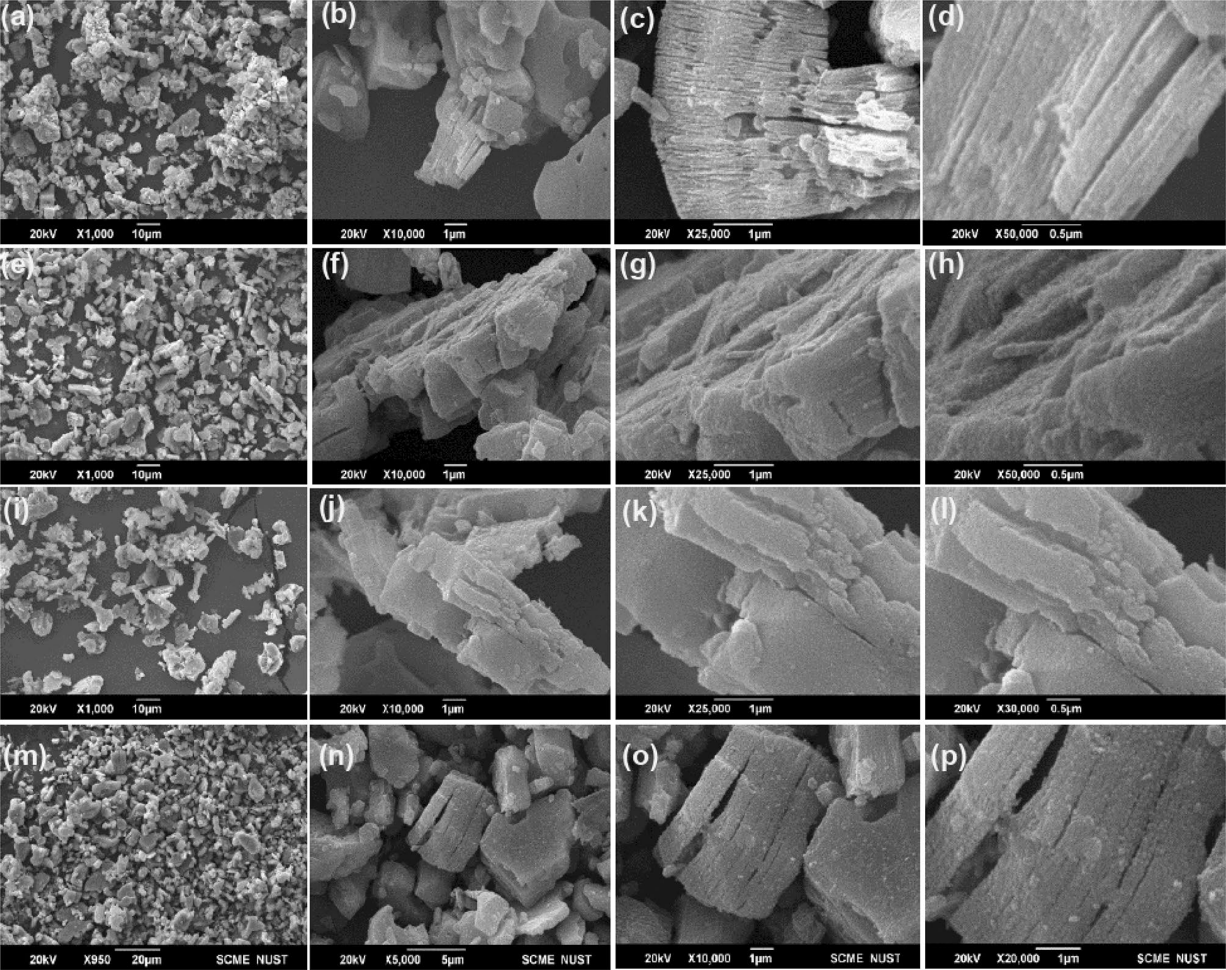
(**a**) SEM micrograph of pure Titanium carbide (Ti_3_C_2_). (**b**) SEM image of Ti_3_C_2_T_x_ at same intensity. (**c**) At 25,000 × zoom accordion-like Ti_3_C_2_T_x_ can be seen (1 µm scale bar) (d) At 50,000 × zoom, the flakes of Ti_3_C_2_, further enlarged. (**e**)–(**h**) SEM micrographs composite 1 at different zoom (scale bars are indicated with each image). (**i**)–(**l**) SEM images of composite 2 at different zooms. (**m**)–(**p**) SEM images of composite 3 at different zooms.

**Figure 5:**
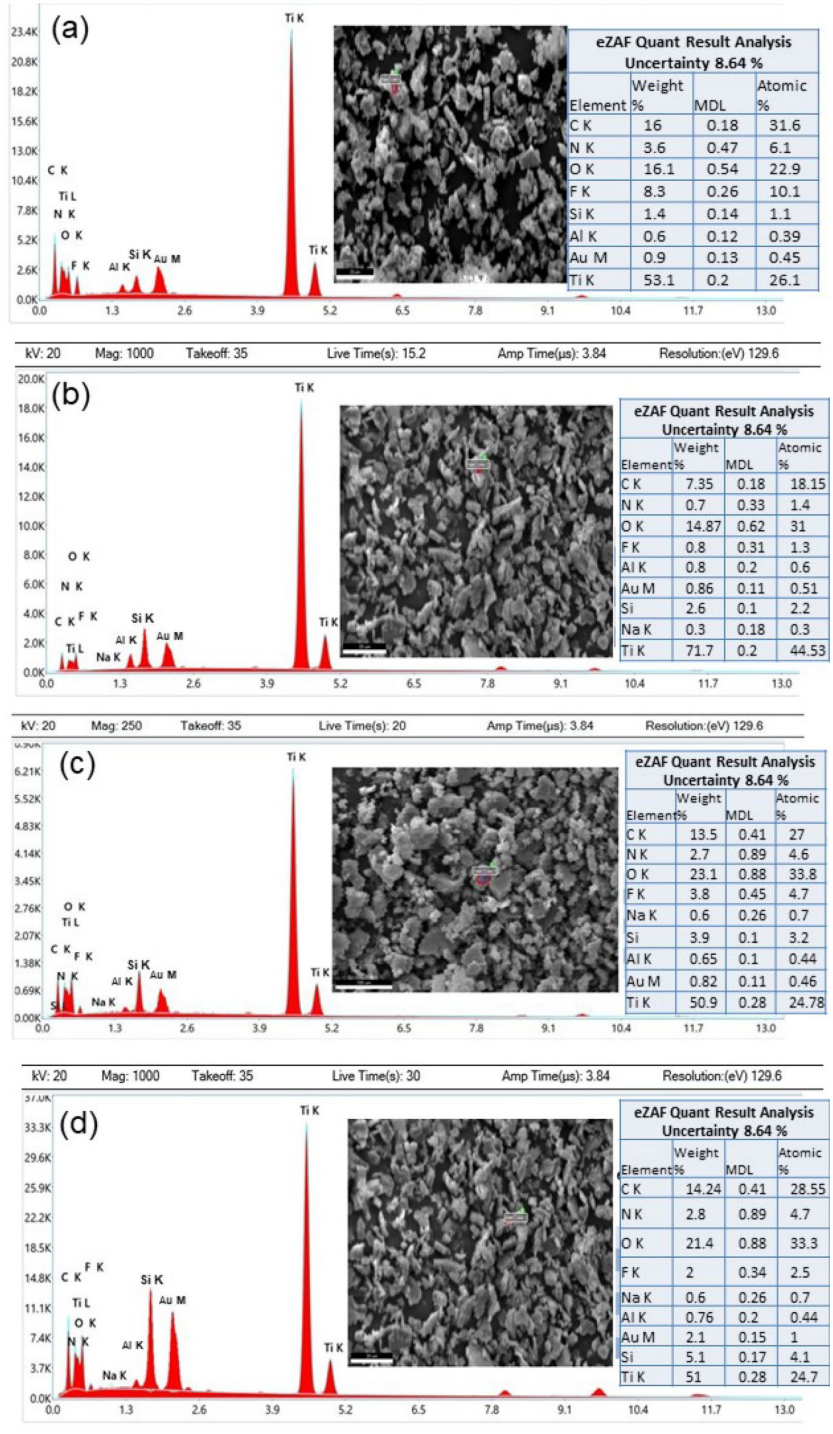
Energy dispersive X-ray (EDX) spectrums and analysis of; (**a**) Ti_3_C_2_; high amount of Titanium (Ti) and carbide (C) with functional group elements are present. (**b**) composite 1_,_ Titanium (Ti) and carbide (C) are present in high quantity while amount of Sodium (Na) and Fluorine (F) is detected. Few functional groups are detected in significant amount. (**c**) Energy dispersive X-ray (EDX) analysis of pure composite 1_,_ the quantitative analysis shows the high amount of Titanium (Ti) and carbide (C), but less amount of Sodium (Na) and Fluorine (F), some functional group elements are also present. (**d**) EDX Spectra of composite 3.

Figure 4a–d show SEM images of the Ti_3_C_2_T_x_ captured at 20 kV, which reveals accordion-like morphology suggesting formation of the delaminated Ti_3_C_2_Tx phase after selective etching of Si. Some delaminated MXene sheets are evident, a predominant presence of stacked lamellar sheets is observed. It is important to note that achieving 100% delamination of MXene sheets is indeed a challenging task, and the observed stacking behavior is not uncommon in practical applications. Figure 5a shows EDX spectrum of Ti_3_C_2_T_x_ MXene, which reveals a considerable amount of Ti and carbon. Various termination group elements such as N, O, and F are present in the MXene. Traces of Si are also present as impurities. Upon careful investigation, we confirm that all peaks in the EDX spectrum, align with the constituents present in the raw materials utilized, however Aluminum (Al-K) and Gold (Au-M) are observed in the range between 1.3 to 2.6 kV may be due to the sample holder. It is crucial to note that these peaks are representative of the elemental composition inherent in the materials used, and after a comprehensive analysis, we can affirm that there is no indication of any additional agent or element beyond the raw material composition and sample holder effects. The SEM and EDX images of Ti_3_C_2_T_x_/NaF composite are shown in Figs. 4 and 5.

Figure 4e–h shows SEM images of Ti_3_C_2_Tx/NaF composite 1. We can also see several NaF spots on the Ti_3_C_2_ surface, which complement XRD results. Figure 5b show EDX spectra of the same sample which reveals significant amount of Ti and carbide, with a modest amount of Na and F, suggesting formation of NaF/Ti_3_C_2_T_x_ composite. Figure 4i–l and m–p show SEM images for composite 2 and composite 3, respectively. All images indicate incorporation of NaF onto the surface of the MXene layers and the results are in agreement with the XRD findings. Figure 5c and Fig. 5d show EDX spectra of the composite 2 and composite 3, respectively. Both figures suggest incorporation of NaF in MXene, wherein that increased Na and F concentration in Fig. 5d suggest higher impregnation of NaF for composite formation.

In Fig. 5a–d, after a meticulous examination, we have substantiated that the peaks discernible in the EDX spectrum align precisely with the constituents inherent in the raw materials employed. It is noteworthy, however, that peaks corresponding to Al-k and Au-M peaks are discerned within the voltage range of 1.3–2.6 kV in all samples. These specific peaks are postulated to arise from the influence of the sample holder. It is imperative to underscore that these identified peaks are indicative of the elemental composition intrinsic to the utilized materials. Following an exhaustive analysis, we can confidently assert that there is no discernible evidence of the presence of any extraneous agent or element beyond the composition of the raw materials and and sample holder. Notably, the observed Al-K and Au-M peaks are attributed to the sample holder effect. Significantly, the ubiquitous nature of the sample holder effect across all samples allows us to discount the impact of these Al-K and Au-M constituents on the overall analysis. Therefore, we can reasonably disregard the influence of these sample holder-related peaks in our assessment of the elemental composition of the materials under investigation.

### Electrochemical performance of NaF-MXene composite

**Figure 6:**
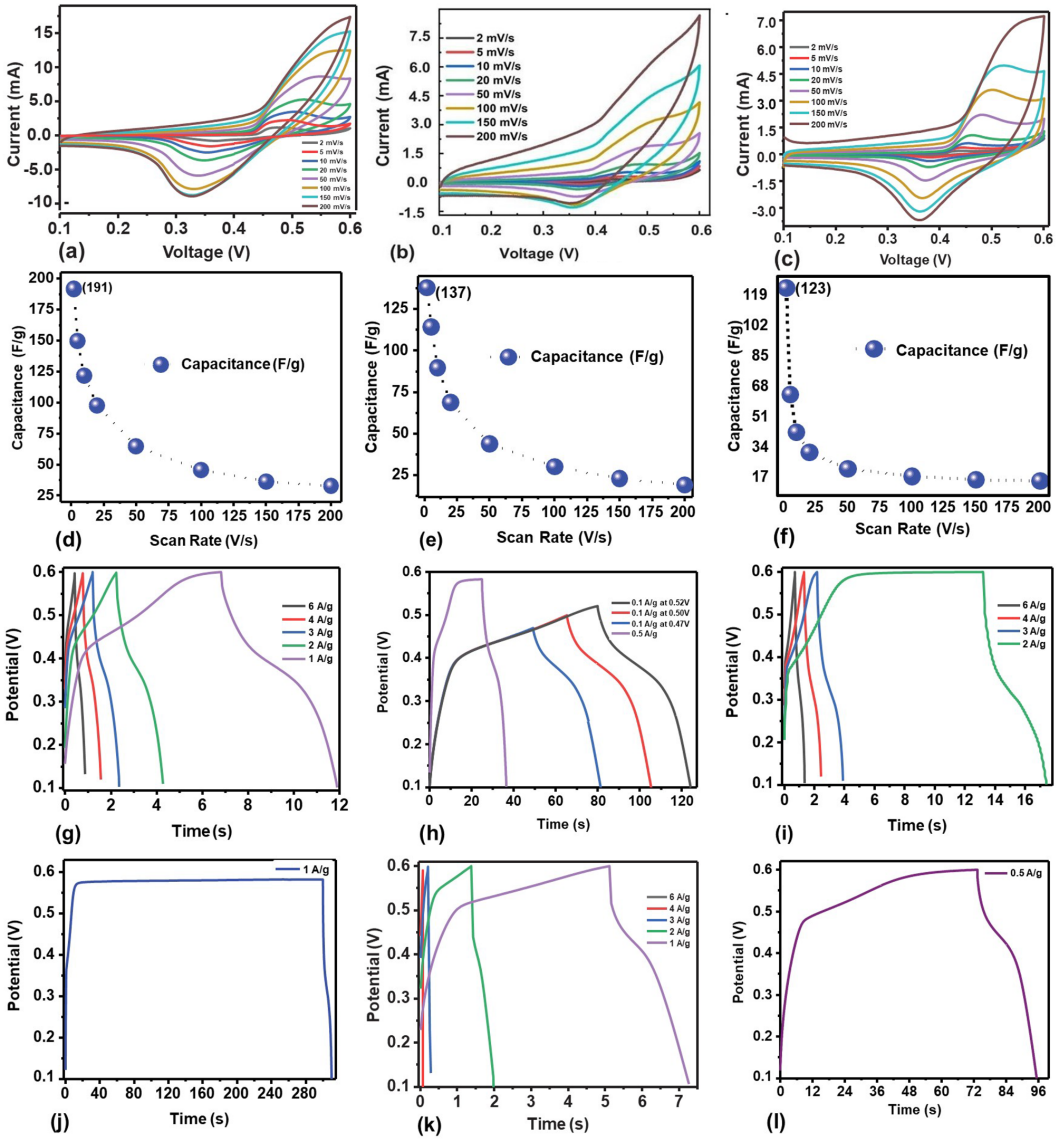
(Three-electrode System). (**a**)–(**c**) Cyclic voltammograms recorded for composite 1 to 3, respectively (The potential is recorded with respect to the reference electrode.) (**d**)–(**f**) Relationship of Scan Rate and Capacitance for composite 1 to 3, respectively. (**g**) GCD curves for composite 1. (h) GCD graph at different potential to avoid saturation region in composite 1. (**i**) GCD curves for composite 2. (**j**) At 1A/g current density, the GCD graph's saturation area appeared below 0.6 V potential for composite 2. (**k**) GCD curves for composite 3. (**l**) At 0.5 A/g current density, the saturation region is visible on the GCD curves of composite 2. GCD curves of (**g**), (**i**) and (**k**) are recorded at different current densities using potential in range of 0.1–0.6 V. The potential values are recorded with respect to the reference electrode.

The electrochemical performance of the fabricated three-electrodes and two-electrodes were perforemed analyzed using cyclic voltammetry (CV) and Electrochemical Impedance Spectroscopy (EIS). In the supplementary file, CV and GCD measurements of composite 1 (Fig. S1), composite 2 (Fig. S2) and composite 3 (Fig. S3) are presented via via two-electrode system. Figure 6a–c, are representative cyclic voltammetry (CV) curves of electrode fabricated from composite 1, composite 2 and composite 3 at different scan rates ranging from 2 to 200 mV/s via thre-electrode system. The analysis of CV was conducted to assess the charge transfer rate at the electrolyte–electrode interface. The nonrectangular-shaped cyclic voltammograms observed in our study indicate the occurrence of faradic behavior, pointing to redox reactions during the charging and discharging cycles. This observation strongly suggests that the battery device exhibits pseudo-capacitor behavior^3,34–37^. The Faradaic current peaks observed across the scanned potential range signify reversible charge and discharge processes taking place at the electrode–electrolyte interface. Redox reactions change high-energy reactants into low-energy products when a battery is coupled to an electrical load. Notably, there is an evident increase in the potential difference between the oxidation and reduction peaks, a phenomenon that becomes more pronounced at higher scan rates. This increase in potential difference is indicative of heightened irreversible and quasi-reversible reactions under faster scan rates. This shift is primarily attributed to the internal resistance of the electrode and the polarization induced by the elevated scan rate. These findings shed light on the dynamic electrochemical processes within the battery, emphasizing the importance of considering factors such as internal resistance and polarization in understanding its performance under different operational codition. The term “battery-supercapacitor”^38,39^ is frequently employed to describe hybrid supercapacitor devices. In conventional batteries and electric double-layer capacitors (EDLCs), there are inherent drawbacks such as low power densities and low energy densities, respectively. These limitations can be effectively addressed through an innovative technology known as hybrid supercapacitors. This technology involves the amalgamation of two distinct technologies—batteries and supercapacitors—into a singular device^40,41^. This integration results in significantly heightened energy storage (Es), increased power density (Ps), the ability for rapid charging/discharging, and an extended cyclic lifespan.

Figure 6d–f, respectively, indicate relationship between scan rate and capacitance for electrode fabricated from composite 1, composite 2 and composite 3, respectively. It is worthy to note that the specific capacitance becomes low at higher scan rates due to phenomenon, like limited ion diffusion, ohmic drop and non-ideal behavior of the electrochemical system.

Contrarily, at lower scan rate, higher specific capacitance is due to enhanced ion diffusion, reduced ohmic drop and extended ionic adsorption on the electrode surface. We observed drop in capacitance from 191 to 20 F/g for scan rate of 2–200 mV/s for device fabricated from composite 1. It was noted that capacitance reduces with the increasing concentration of NaF. For example, highest capacitance for composite 1 was 191 F/g, which was dropped to 137 F/g in case of composite 2. Smaller amount of NaF improve capacitance, however increased amount of NaF resulted in lower capacitance. The charge storage phenomenon for the developed electrodes were studied using Galvanostatic Charging Discharging (GCD). Figure 6g–l, show GCD curves for electrode fabricated from composite 1, 2 and 3.

The plots show the charging and discharging at different gravimetric current densities within voltage range of 0.1–0.6 V. They exhibited symmetrical and triangular shaped charge discharge profiles suggest characteristic pseudocapacitive behavior of the electrode material. For stable operational current density range, we tested device at different values such as 6 A/g to 4, 3, 2, 1, 0.5, 0.1 A/g. The charging and discharging process was completed within 1 s at a current density of 6 A/g. On the other hand, charging and discharging time significantly increased at 0.5 A/g; however, the device entered into a saturation region. This can be avoided by limiting voltage window from 0.6 to 0.5 V. As illustrated in Fig. (h), (j), and (l), saturation region disappeared as soon as the voltage window was reduced. Energy density and power density were calculated using Eqs. (2, 3). For example, highest energy density and power density of 6.63 Wh/g and 54.24 W/g were calculated for composite 1. Specific charge capacity was also calculated for all samples and we observed that composite 1 has highest specific charge capacity of 48.8 F/g at current density of 1 A/g. Composite 2 showed the specific charge capacity of 28.27 F/g and composite 3 gave us a specific charge capacity of 12.2 F/g at current density of 1 A/g. Highest specific charge capacity, energy density and power density for composite 1 is in accordance with CV measurements and thus enhance our claim of composite 1 being the best optimized electrode material. Overall, the measured values of both CV and GCD are same using three-electrodes and two-electrodes studies.

**Figure 7:**
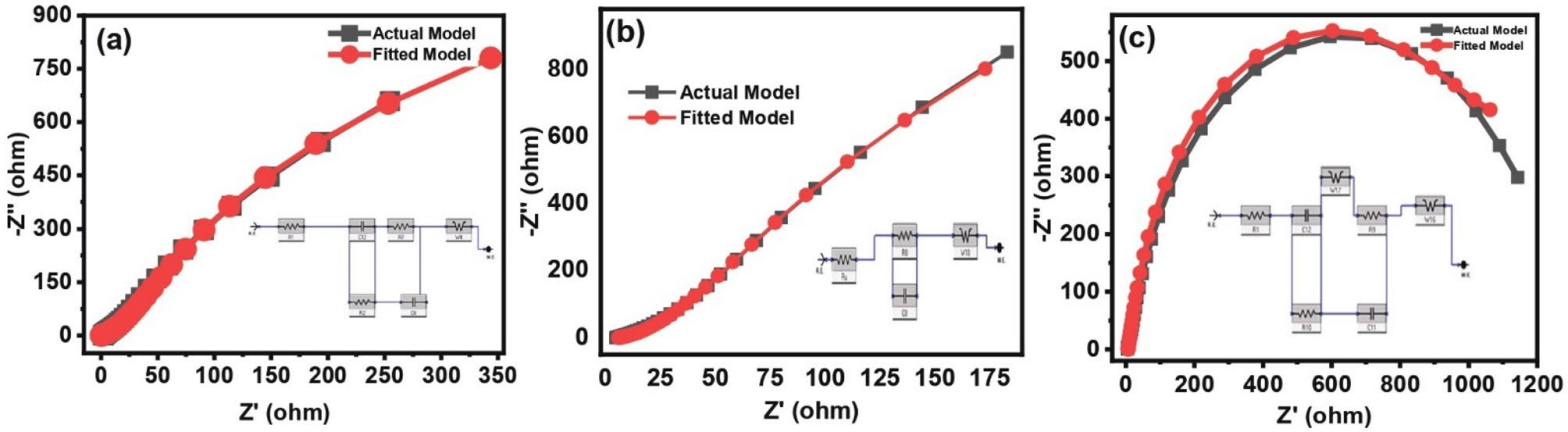
(Three-electrode System), EIS curves of experimental data and fitted model of; (**a**) Composite 1. (**b**) Composite 2. (**c**) Composite 3.

Electrochemical Impedance Spectroscopy (EIS) is an important tool which offers valuable insights into ion transfer and resistive as well as capacitive properties of the electrode. Figure 7, shows the EIS Nyquist plots for composite 1, 2 and 3, which were measured in a frequency range from 100 kHz to 10 MHz. Real and imaginary impedance are denoted by Z′ and Z″ ohm, respectively, whereas left and right portions of the curves refer to higher and lower frequencies, respectively.

The intersection of plots on the real axis at higher frequencies indicates the internal resistance of the device, which include electrolyte resistance, electroactive material’s intrinsic resistance, and contact resistance^42^. Similarly, semicircle's diameter at high frequencies indicates faradic charge transfer resistance (R_ct_) of the redox reactions, whereas, any inclined line at low frequencies corresponds to Warburg impedance^43^. Furthermore, the region of the graph with a 45° slope at higher frequency region is attributed to higher diffusion rate. Figure 7a–b, clearly show a slope of 45° at higher frequency region which corresponds to higher diffusion process in the composite with lower concentration of NaF leading to the higher capacitance. The result is in good agreement with CV results. The fitted model was applied to the actual model using Randle's model. For this purpose, an equivalent circuit using an ESR, a resistor, a capacitor, and a Warburg resistor was created as illustrated in the inset of Fig. 7. The increasing size of semicircle at the higher frequency region with decreasing content of NaF indicate lower charge transfer resistance at lower NaF.

The results presented in Table 1 show that Composite-1 with a composition of 1% NaF and 99% Ti3C2Tx exhibited the highest specific capacitance, energy density, and power density compared to Composite-2 and Composite-3. Composite-1 (1% NaF/99% Ti3C2Tx) demonstrated superior electrochemical performance compared to Composite-2 and Composite-3, as evidenced by higher specific capacitance, energy density, and power density. The optimal composition of 1% NaF in conjunction with 99% Ti3C2Tx likely led to effective surface modification, enhancing conductivity, ionic mobility, and structural integrity. This composition may have facilitated optimized electrolyte interaction and generated synergistic effects, resulting in Composite-1's outstanding electrochemical properties. However, further investigations, including detailed characterization and additional experimentation, are warranted to precisely elucidate the mechanisms contributing to the observed performance differences among the composite samples.

**Table 1:** Specific capacitance, energy density and power density of composite samples (Three-electrode System).

No	Concentration	Specific capacitance (F/g)	Energy density (Wh/g)	Power density (W/g)
1	Composite-1(1%NaF/99%Ti_3_C_2_T_x_)	191	6.63	54.24
2	Composite-2(3%NaF/97%Ti_3_C_2_T_x_)	137	4.76	25.96
3	Composite-3(5%NaF/95%Ti_3_C_2_T_x_)	123	4.27	23.64

**Figure 8:**
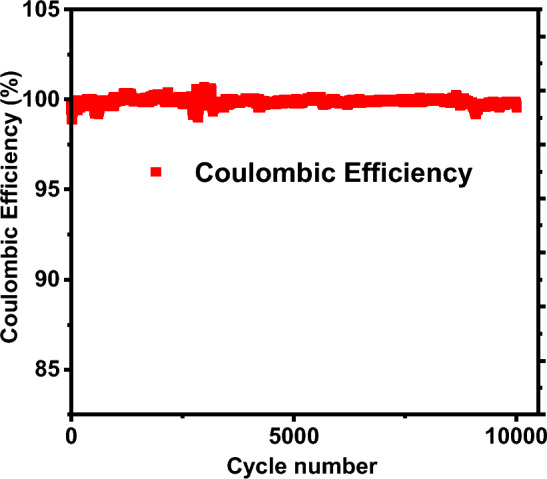
Stability test of Composite-1(1%NaF/99%Ti3C2Tx) electrode showing the coulombic efficiency for 10,000 cycles.

The stability assessment of the prepared Composite-1 is depicted in Fig. 8, conducted under a constant current density of 0.6 mAcm^(-2). Given the prolonged duration of the evaluation spanning several days, the durability and stability of the device were scrutinized under the specified minimum current density conditions. Despite the extended testing period, the electrode demonstrated a commendable performance, maintaining a coulombic efficiency of over 90%. Coulombic efficiency, representing the ratio of the charges extracted from the supercapacitor relative to the charge required for restoring the initial capacity, serves as a crucial metric for assessing the electrochemical stability of the device. Notwithstanding minor deviations, the observed > 90% coulombic efficiency underscores the robustness and enduring stability of Composite-1 under the specified operational conditions. This outcome substantiates the suitability of the composite material for prolonged and reliable use in energy storage applications.

The specific capacitance is a valuable metric for assessing the performance of electrode materials in certain applications, in the context of battery-grade materials, other relevant performance indicators, such as energy density, power density are also measured as depicted in Table 1 and life cycle presented in Fig. 8. We have included relevant metrics that better align with its intended use in hybrid battery-supercapacitor applications.

## Conclusion

We synthesized and characterized a novel Ti_3_C_2_T_x_/NaF composite through hydrothermal process for potential energy storage applications. Three distinct composite samples were synthesized, each with varying concentrations of NaF (1%, 3%, and 5%). The electrochemical properties and behavior of these composites as supercapacitor electrodes were explored. The structural analysis of the composites was carried out using X-ray diffraction (XRD) and scanning electron microscopy (SEM). XRD patterns of the composites exhibited shifted peaks and new peaks associated with the presence of NaF, confirming the successful formation of the Ti_3_C_2_Tx/NaF composite. SEM images showcased the accordion-like morphology of Ti_3_C_2_Tx, indicating successful delamination, while NaF spots were observed on the surface, validating the incorporation of NaF in the Ti_3_C_2_Tx. Electrochemical performance was evaluated through cyclic voltammetry (CV), galvanostatic charging-discharging (GCD), and electrochemical impedance spectroscopy (EIS). Our I-V curves demonstrated characteristics reminiscent of batteries, additionally, the GCD analysis provided further validation of the supercapacitor nature of the composites, showcasing rapid and symmetrical charge–discharge profiles. This dual observation solidified the hybrid battery-supercapacitor nature inherent in our composite samples, highlighting a versatile energy storage system that combines the endurance of batteries with the rapid charge–discharge capabilities of supercapacitors. The energy density and power density of the composites were calculated, with composite 1 exhibiting the highest values of 6.63 Wh/g and 54.24 W/g, respectively. Furthermore, composite 1 showed considerably a specific capacitance of up to 191 F/g at a scan rate of 2 mV/s in KOH electrolyte. The presence of NaF influenced the charge transfer resistance and diffusion rate, with lower NaF concentrations resulting in improved charge transfer characteristics. The tailored properties of these composites, influenced by NaF concentration, offer promising avenues for advanced energy storage solutions. This hybrid approach ensures a dynamic energy storage solution that combines the endurance of a battery for extended durations with the rapid response and high-power performance of a supercapacitor, resulting in an optimized system capable of meeting diverse energy storage requirements.

### Supplementary Information

Supplementary Figures.

## Data Availability

The datasets used and/or analyzed during the current study are available from the corresponding author on reasonable request.
