# Supplementary material for: 2-Dimensional Ti3C2Tx/NaF nano-composites as electrode materials for hybrid battery-supercapacitor applications
Source: Sci Rep. 2024 Jan 18;14:1654. doi: 10.1038/s41598-024-52280-4 (PMC10796376; doi:10.1038/s41598-024-52280-4)
Supplement: Supplementary file 1 — Supplementary Figures. [file 41598_2024_52280_MOESM1_ESM.docx]

Supplementary:

**2-Dimensional Ti_3_C_2_T_x_/NaF Nano-composites as Electrode Materials for hybrid Battery-Supercapacitor Applications**

**M. Bilal Riaz^1^, Danish Hussain^2^, Saif Ullah Awan^1†^, Syed Rizwan^3^, Sana Zainab^1^, Saqlain A. Shah^4^**

1. Department of Electrical Engineering, College of Electrical and Mechanical Engineering, National University of Sciences and Technology (NUST), Islamabad 44000, Pakistan.
2. Department of Mechatronics Engineering, NUST College of Electrical and Mechanical Engineering, National University of Sciences and Technology (NUST), Islamabad 44000, Pakistan
3. Physics Characterization and Simulation Lab (PCSL), Department of Physics, School of Natural Sciences (SNS), National University of Sciences and Technology (NUST), Islamabad 44000, Pakistan.
4. Department of Physics, Forman Christian College (University), Lahore, Pakistan

**^†^** Corresponding Author: [saifullahawan@ceme.nust.edu.pk](mailto:saifullahawan@ceme.nust.edu.pk) [ullahphy@gmail.com](mailto:ullahphy@gmail.com)

**Figure S1** (Two-electrode System)**:** Using two-electrode configurations **(a)** CV graph of 1% composite concentration of Ti_3_C_2_/NaF **(b)** Scan Rate vs Capacitance **(c, d)** GCD graph at different current density

**Figure S2** (Two-electrode System)**:** Using two-electrode configurations **(a)** CV graph of 3% composite concentration of Ti_3_C_2_/NaF **(b)** Scan Rate vs Capacitance **(c)** GCD graph at different current density

**Figure S3** (Two-electrode System)**:** Using two-electrode configurations **(a)** CV graph of 5% composite concentration of Ti_3_C_2_/NaF **(b)** Scan Rate vs Capacitance **(c, d, e)** GCD graph at different current density
